# The Effects of Intensive Weight Reduction on Body Composition and Serum Hormones in Female Fitness Competitors

**DOI:** 10.3389/fphys.2016.00689

**Published:** 2017-01-10

**Authors:** Juha J. Hulmi, Ville Isola, Marianna Suonpää, Neea J. Järvinen, Marja Kokkonen, Annika Wennerström, Kai Nyman, Markus Perola, Juha P. Ahtiainen, Keijo Häkkinen

**Affiliations:** ^1^Department of Biology of Physical Activity, Neuromuscular Research Center, University of JyväskyläJyväskylä, Finland; ^2^Department of Physiology, Faculty of Medicine, University of HelsinkiHelsinki, Finland; ^3^Department of Health Sciences, University of JyväskyläJyväskylä, Finland; ^4^Department of Physical Education, University of JyväskyläJyväskylä, Finland; ^5^Genomics and Biomarkers Unit, Department of Health, National Institute for Health and WelfareHelsinki, Finland; ^6^Institute for Molecular Medicine Finland and Diabetes and Obesity Research Program, University of HelsinkiHelsinki, Finland; ^7^Central Hospital of Central FinlandJyväskylä, Finland; ^8^The Estonian Genome Center of the University of TartuTartu, Estonia

**Keywords:** fat loss, exercise, nutrition, fitness, body composition, sex hormones, thyroid hormones

## Abstract

Worries about the potential negative consequences of popular fat loss regimens for aesthetic purposes in normal weight females have been surfacing in the media. However, longitudinal studies investigating these kinds of diets are lacking. The purpose of the present study was to investigate the effects of a 4-month fat-loss diet in normal weight females competing in fitness-sport. In total 50 participants finished the study with 27 females (27.2 ± 4.1 years) dieting for a competition and 23 (27.7 ± 3.7 years) acting as weight-stable controls. The energy deficit of the diet group was achieved by reducing carbohydrate intake and increasing aerobic exercise while maintaining a high level of protein intake and resistance training in addition to moderate fat intake. The diet led to a ~12% decrease in body weight (*P* < 0.001) and a ~35–50% decrease in fat mass (DXA, bioimpedance, skinfolds, *P* < 0.001) whereas the control group maintained their body and fat mass (diet × group interaction *P* < 0.001). A small decrease in lean mass (bioimpedance and skinfolds) and in vastus lateralis muscle cross-sectional area (ultrasound) were observed in diet (*P* < 0.05), whereas other results were unaltered (DXA: lean mass, ultrasound: triceps brachii thickness). The hormonal system was altered during the diet with decreased serum concentrations of leptin, triiodothyronine (T3), testosterone (*P* < 0.001), and estradiol (*P* < 0.01) coinciding with an increased incidence of menstrual irregularities (*P* < 0.05). Body weight and all hormones except T3 and testosterone returned to baseline during a 3–4 month recovery period including increased energy intake and decreased levels aerobic exercise. This study shows for the first time that most of the hormonal changes after a 35–50% decrease in body fat in previously normal-weight females can recover within 3–4 months of increased energy intake.

## Introduction

Many persons lead an active lifestyle aiming to lose body fat with minimal muscle loss for aesthetic or performance purposes. An extreme example of these individuals are competitors in different aesthetic and/or weight class sports (Sundgot-Borgen et al., 2013). These fat loss regimens are very popular and are shown regularly in the media in a similar way as diets for the obese are (Fothergill et al., 2016). Although, worries have arisen about the negative consequences of these diets on, for example, the female hormonal system, comprehensive longitudinal weight-loss studies investigating these diets are lacking on this area, especially in normal-weight females. This lack of studies is probably due to ethical reasons and practical constraints as it is not ethical to conduct a randomized controlled trial (RCT) with normal weight individuals so that the participants would diet as heavily as needed in these kinds of situations.

A classic Minnesota starvation study in the 1940s investigated the effects of prolonged and extreme dieting in young previously normal weights males (Keys et al., 1950; Dulloo et al., 1996). This type of a long-term semi-starvation experiment would no longer be possible in civilized countries and thus only much shorter semi-starvation studies have been conducted, mainly in males (Alemany et al., 2008; Henning et al., 2014; Müller et al., 2015). Nevertheless, cross-sectional and longitudinal refeeding studies have been conducted in females with low energy availability such as anorexia nervosa or with the female athlete triad. These studies have shown that prolonged undernutrition is often associated with low fat and lean mass, decreased bone mineral content, and other physiological and psychological changes from which recovery can be very slow and difficult (Sundgot-Borgen et al., 2013). It is not known how well a normal weight female body can recover from an energy deficit.

There is a lack of studies in females even though they often take part in weight reduction ending up in a rather large energy deficit. For this research question, female fitness competitors are important as they voluntarily conduct a prolonged heavy diet concurrent to participating in a large amount of exercise. The aim of these competitors is to achieve an aesthetic appearance with symmetry, balance, and muscle “definition” that is accomplished by low fat mass. These competitors usually compete and thus diet once per year. Their routine includes a 2–5 month progressive diet ending up in a state of low energy intake, which is usually achieved mainly by decreasing carbohydrate and/or fat intake with maintenance of high protein. Furthermore, exercise volume is increased to reduce fat mass effectively while maintaining muscle mass (Helms et al., 2014, 2015). The diet is typically followed by a recovery period, during which the competitors increase their energy intake back to baseline. This is quite a contrast to overweight individuals, who try to maintain their weight loss, although only rarely that goal is achieved (Fothergill et al., 2016). Previous weight loss studies with fitness athletes or bodybuilders have often been case studies using male bodybuilders (Rossow et al., 2013; Kistler et al., 2014; Robinson et al., 2015) or they have focused only on body composition, muscle strength (Sandoval et al., 1989; Bamman et al., 1993; van der Ploeg et al., 2001), or psychology (Newton et al., 1993). Thus, physiology, including the hormonal system, has not yet been comprehensively investigated in a larger group of individuals following a diet that aims to achieve very low levels of fat mass.

In the present study voluntary fitness-competitors and their controls are used in a unique research model to investigate the physiological effects of a demanding 3–4 month period of dietary energy restriction concurrent with a large amount of exercise aiming to achieve prolonged negative energy balance. It is also asked whether a similar 3–4 month period of increased energy intake is enough to restore endocrine function and body composition. We hypothesized that (1) fitness-competitors are able to decrease their body mass mainly by decreasing fat mass and that (2) the endocrine system is altered, approaching levels that are typically considered unhealthy if maintained for longer periods of time, and that (3) the hormonal levels are increased back to the baseline together with body weight during the recovery period.

## Methods

### Participants

A total of 184 healthy, physically active young females, recruited by web page and social media advertisements and who claimed to meet the inclusion criteria volunteered for the study. Out of the females who fulfilled the preliminary criteria, 63 participants were planning on competing under the International Federation of Bodybuilding and Fitness (IFBB) in the year of the study and, thus, would diet accordingly. In addition, a total of 121 volunteers were aiming not to diet now, but would probably diet later in their life or at least try to maintain a fitness lifestyle. An online pre-study questionnaire was sent to the available 44 diet- and 70 randomly chosen control group candidates that fulfilled the preliminary requirements for the study (see below). Females, who were diagnosed with chronic diseases or prescribed medications such as thyroxine, but excluding contraception, and who were younger than 20 or older than 38 years old, whose BMI was below 20 or above 27, or who did not have at least 2 years of resistance training experience were excluded from the study. As a result, the diet group competing in the autumn of 2015 consisted of 30 volunteers. We chose these exclusion criteria because our aim was to investigate normal-weight healthy previously trained females. An equal number of control participants were quasi-randomized by matching based on their age, height, weight, and training experience reported on the pre-study questionnaire. The participants selected for the study filled in an additional questionnaire that was subsequently reviewed by the physician of our study to confirm that they did not meet the exclusion criteria relating to health.

All of the diet participants were IFBB amateur fitness competitors aiming to lose fat, but maintain their muscle mass in a sport that is tested for prohibited performance enhancing drugs. Out of these participants, 17 were bikini fitness and 9 body fitness competitors and 1 was a fitness competitor. These groups were very similar at the baseline and with regard to the changes during diet (body composition and hormones, data not shown).

The subjects were given comprehensive explanations regarding the study design, protocols, and possible risks. This study was carried out in accordance with the recommendations of Ethical Committee at the University of Jyväskylä with written informed consent from all subjects. All subjects gave written informed consent in accordance with the Declaration of Helsinki. The protocol was approved by the Ethical Committee at the University of Jyväskylä.

### Study design

The study included 3 test days at the laboratory: baseline testing before the diet or the control period started (Pre), after the diet (Mid), and after a refeed recovery period (Post) during which the participants were advised to continue their training regimen, but to stop dieting. The control participants were advised to maintain their activity levels and nutrient intake throughout the study. The participants were given identification numbers and the measurement team was blinded so that they did not know into which group participants belonged to.

**Figure 1:**
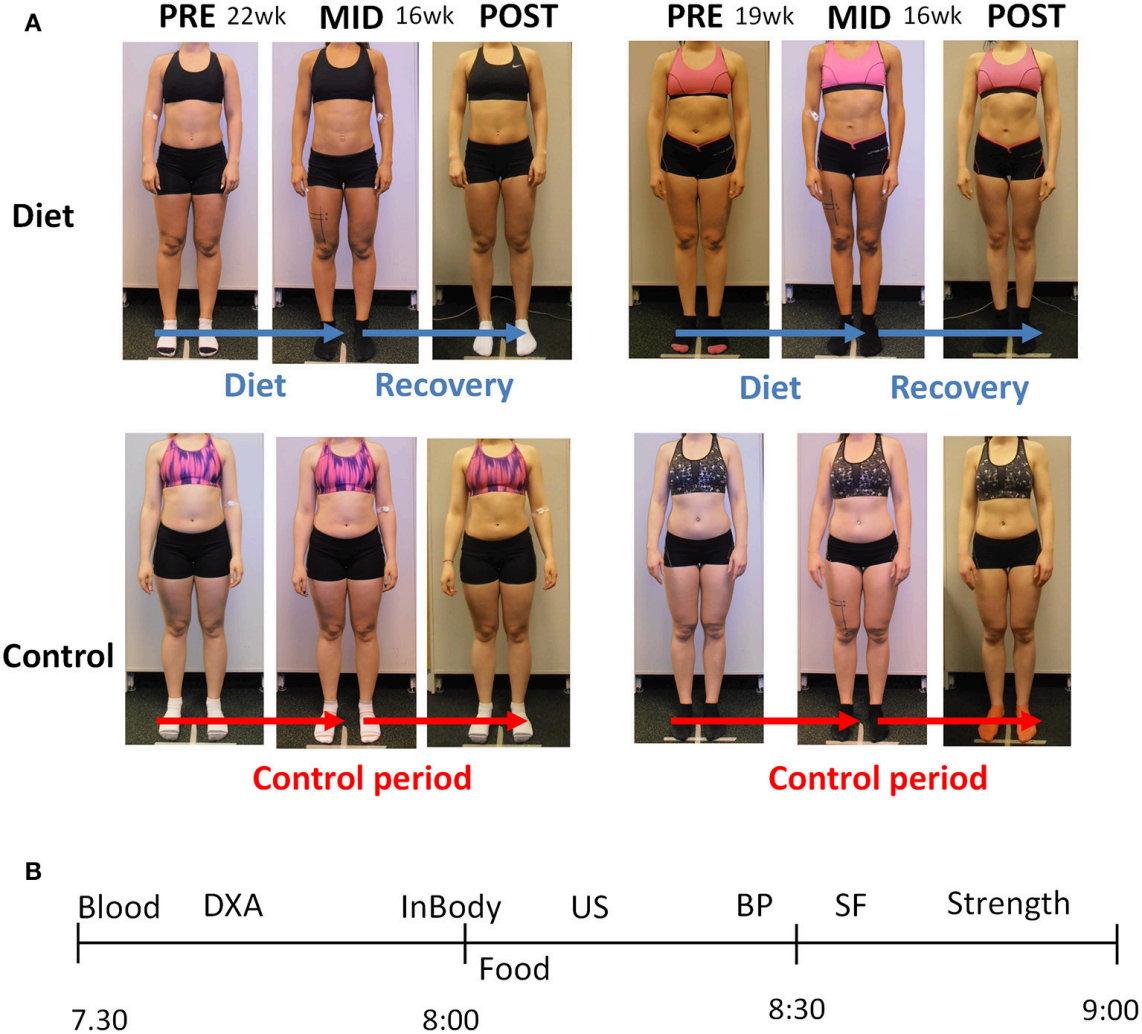
**(A)** The experimental design of the study. Two representative participants are shown from each group. The pre to mid time period lasted ~20 weeks during which the participants decreased their energy intake and the amount of exercise (see the Results Section), whereas the controls maintained their activity levels and nutrient intake. The mid to post period lasting ~18 weeks was a recovery period with increased energy intake back toward the baseline levels in the diet participants, whereas the controls maintained their energy intake and exercise levels (see the Results Section). **(B)** The measurement day example for each participant. The x-axis depicts AM-time (morning). Blood, blood sample; DXA, Dual-energy X-ray absorptiometry; InBody, bioelectrical impedance; Food, breakfast; US, ultrasound; BP, blood pressure; SF, skinfolds; Strength, muscle strength measurements.

Out of the 30+30 participants eventually 30 volunteers that would follow the diet (referred to as “diet group” from here on) and 29 controls arrived to our baseline testing. During the study, nine females (3 from the diet group and 6 controls) dropped out. All three drop-outs in the diet group stopped dieting due to failing to follow the diet program whereas controls either were not able to follow the control period or for some unknown reason finished the study. This resulted in 50 participants (27 dieters and 23 controls) overall finishing the whole study. The diet group (*n* = 27) was on average 27.2 ± 4.1 years old and the controls 27.7 ± 3.7 years old. The length of the diet-period for the diet group was 19.8 ± 3.6 weeks while the recovery period was 17.5 ± 2.6 weeks. The length of the respective control periods were 22.4 ±5.0 and 19.2 ± 5.3 weeks, respectively. Figure 1A depicts the study design and two females from each group.

For 15 of the competitors, this was their first competition diet and the rest of the diet group (*n* = 12) had been dieting for competitions between 1 and 4 times. Three of the 27 of the diet group participants were competing at the world championship level, while the rest competed in the Finnish National championships or in the qualification rounds.

### Three test days

The participants came to the laboratory from all over the Finland. If they traveled >50 km to our laboratory, they were provided with a hotel room for the night prior to testing. Figure 1B depicts the measurement day of the participant in the laboratory. The participants came to the tests after 8 h of fasting and the first measurements (blood sampling, DXA, bioimpedance) were conducted before a quick standardized low-fat breakfast (a protein-drink, a protein bar and a medium-sized apple/banana: in total ~47–48 g proteins, ~72–80 g carbohydrates, ~6 g fat). Thereafter, ultrasound, skinfolds, blood pressure, and muscle strength measurements were conducted. The breakfast was especially important in the measurements after the diet since some of the participants were in a large energy deficit and potentially not able to perform, for example, muscle strength testing in a reliable and safe manner. The mid measurement day after the diet was conducted the morning after the competition.

All the measurements were conducted at the same time (always within max ±1 h) due to the importance of standardizing the time of the day of measurements. The control group participants were measured on the same days as the competitors. The same researcher/research assistant was always responsible for conducting the measurements and analysis to avoid interobserver/-analyzer variability. The participants were asked to sleep for at least 8 h during the preceding night and were required to refrain from strenuous physical activity for at least 24 h.

### Resistance and aerobic training

The resistance training background of the participants was 3.5 ± 1.4 years in the diet group and 3.1 ± 1.1 years in the control group. The participants trained with their own training programs and they were asked to provide their training diaries throughout the study period. The training frequency, intensity and volume were calculated from the diaries. Total exercise metabolic equivalents in hours (MET-hours) were calculated based on recommendations (Ainsworth et al., 2000).

Split routines were used for resistance training by all competitors in the diet group meaning that they focused on single muscle groups per session as is often the case also in bodybuilders (Hackett et al., 2013). The main muscle groups trained included thighs, hamstrings, buttocks, chest, shoulders, arms, upper and lower back, calves, and abdominals. Dividing training into separate body parts per session did not differ significantly throughout the training. At baseline the 3-, 4-, 5-, and 6-split training was used by 3, 10, 13, and 1 of the 27 participants, respectively, while the same numbers were during the diet on average 5, 8, 14, and 0 and during the recovery period 7, 8, 12, and 0. In addition, the competitors also practiced their posing routines. Training sessions lasted between 40 and 90 min.

Aerobic training for the participants was almost uniquely either high-intensity interval training (HIT) with bicycle, crosstrainer, or other gym equipment or both HIT and steady-state low to medium intensity aerobics (usually walking/running or with crosstrainer). During the competition week the participants did not report doing HIT, but instead lower intensity aerobics. Typical HIT-exercise was 10–25 min in total including high intensity 15–45 s intervals with 30–60 s of recovery between the sets. Steady state lower intensity aerobics was typically 30–60 min in duration. Part of the females completed their aerobic training mainly together with their resistance exercise workouts while most of the participants completed also separate aerobic workouts, especially during the diet.

At the last week of a typical fitness or bodybuilding diet there is a tapering period during which total training load is typically slightly decreased and carbohydrate and total energy intakes are increased toward the baseline levels. This is conducted to replenish muscle glycogen stores and, thus, prevent an artificial decrease in muscle size that occurs with low carbohydrate diets as ~2.7 g of water per each gram of glycogen is stored in skeletal muscle.

### Nutrient intake

The participants maintained their diet during the diet phase. About 50% percent of the participants reported all their meals to the investigators. The rest of the participants had a more flexible diet or did not share all the details of their diet, but, instead reported representative days throughout the diet. The controls kept their dietary diary over 3 weekdays and 1 weekend day at the baseline, in the middle of the study and during the last part of the study. Nutrients provided by nutritional supplements were included in the analysis. The food diaries were analyzed by nutrient analysis software (Aivodiet, Flow-team Oy, Oulu, Finland).

### Body composition

#### DXA

Body composition was estimated by Dual-energy X-ray absorptiometry (DXA, Lunar Prodigy Advance, GE Medical Systems—Lunar, Madison WI USA) using methods similar to Hulmi et al. (2015) in a fasted state. Participants were tested on their back in a supine position on the DXA table with their arms at their sides and feet together with minimal clothing (i.e., a pair of shorts). The legs were secured by non-elastic straps at the knee and ankles, and the arms were aligned along the trunk with the palms facing the thighs. All metal objects were removed from the participant before the scan. Analysis (using enCORE 2005, version 9.30 and Advance 12.30) provided total, lean (bone-free), bone, and fat masses. The android region is the area between the ribs and the pelvis within the trunk region (the upper part of the trunk) and correlates with visceral fat measures (Hill et al., 2007; Miazgowski et al., 2014). High levels of visceral fat mass are strongly associated with metabolic abnormalities (Kang et al., 2011). The gynoid area defined by the software is a region including the sex organs and lower-part of the hips (Miazgowski et al., 2014). In a previous study in our laboratory the intraclass correlation coefficient (ICC) for the body composition measures were 0.786–0.975 (Schumann et al., 2014).

#### Bioimpedance

After an overnight fast, body fat percentage, fat, and lean masses were measured by bioelectrical impedance using an InBody720 machine with a multifrequency current (Seoul, Korea).

#### Skinfolds

Skinfold thicknesses were analyzed with Harpender calibers from four sites: biceps, triceps, subscapular, and suprailiac and replicated at least three times. Fat percentage was calculated with a formula (Durnin and Womersley, 1974) while total fat mass and fat-free masses were calculated by subtraction from body mass.

### Ultrasound for muscle cross-sectional area and thickness and subcutaneous fat thickness

Cross-sectional area (CSA) of the vastus lateralis muscle was examined at the mid-thigh by extended field of view mode using a B-mode axial plane ultrasound (model SSD-α10, Aloka, Tokyo, Japan) using a 10 MHz linear-array probe (60 mm width) with the extended-field-of-view mode (23 Hz sampling frequency). The CSA of the vastus lateralis muscle was determined from two levels: the first was exactly 40% from the superior point of patella toward anterior superior spina iliaca and the second 2 cm distally from the first level. The thickness of subcutaneous fat in the thigh was examined from the 40% line mentioned above, but at the medial-lateral axis at the border of vastus lateralis and rectus femoris. The thickness of triceps brachii muscle and subcutaneous fat were measured at exactly the midpoint between medial epicondyle and acromion. A customized convex-shaped probe support coated with water-soluble transmission gel was used to assure a perpendicular measurement and to constantly distribute pressure on the tissue. For CSA, the transducer was moved manually from medial to lateral along a marked line on the skin. Three images were scanned from each lines (CSA) and measurement point (thicknesses). CSA and thicknesses were analyzed manually using ImageJ software (version 1.44p; National Institutes of Health, Bethesda, MD). The average values were used for the statistical analyses. The panoramic ultrasound method has been shown by us to be very reliable and valid against magnetic resonance imaging (MRI) to detect resistance training-induced change in muscle CSA in our laboratory, e.g., ICC > 0.9 and high limits of agreement by Bland Altman method (Ahtiainen et al., 2010). Also for subcutaneous fat thickness intra-assay CV and reliability for this method are high (Müller et al., 2016).

### Maximal and explosive strength

In the beginning of the actual measurements, the participants were carefully familiarized with the test procedures and techniques. The participants had a general warm up (body-weight 1- and 2-legged squats and hand rotations) after which several warm-up trials were performed on each test device. A horizontal leg press extension dynamometer (custom built: Department of Biology of Physical Activity, University of Jyväskylä, Jyväskylä, Finland) was used to determine maximal isometric bilateral leg press force (maximal voluntary contraction, MVC). In the leg press, the participants were seated with a hip and knee angle of 110° and 107°, respectively. Maximal leg extension force was analyzed by a customized script (Signal 4.04, Cambridge Electronic Design, UK). Maximal isometric bilateral bench press for the upper-body strength (MVC) was measured in a smith-machine and the bar was locked to the position with the elbow angle of 90° (Ojasto and Häkkinen, 2009). The subject was in a supine position on the bench and pushed against the fixed bar. The test position including the wideness of the grip was standardized throughout the study. The maximum force was measured by the force plates under the bench using a customized script (Signal 4.04, Cambridge Electronic Design, UK). In these isometric tests, the participants were instructed to produce maximal force on verbal command and to maintain their maximal force plateau for 3–4 s while being verbally encouraged. Maximal explosive strength of the hip and knee extensors was measured using a vertical counter movement jump (Komi and Bosco, 1978) on a custom-built infrared contact mat (Department of Biology of Physical activity, University of Jyväskylä). The height of rise of the center of gravity was calculated from the flight time. The subjects were instructed to perform a quick and explosive countermovement and to jump as high as possible while their hands were on the hips throughout the action. In each strength test 3 maximal trials were conducted with recovery periods of 1 min in the isometric tests and 0.5 min in the jump test. Up to two additional trials were performed if the result during the last trial was greater by 5% compared with that during the previous attempt. The trial with the highest result was used for statistical analysis.

### Blood pressure and heart rate

The participants were seated in a quiet room and after resting their blood pressure and heart rate (pulse) was measured using calibrated Omron M6 automatic blood pressure monitor with appropriate cuff size (Omron R6, Omron Healthcare, Kyoto, Japan). The measurements were conducted three times and the average was selected as the final result.

### Venous blood sampling and analysis

Venous blood samples were taken from the antecubital vein into serum tubes (Venosafe; Terumo Medical Co., Leuven, Hanau, Belgium) using standard laboratory procedures. Whole blood was immediately analyzed (Sysmex XP 300 analyzator Sysmex Inc, Kobe, Japan) for hemoglobin and hematocrit. Blood samples for hormone analysis were stored in room temperature for 30 min, after which they were centrifuged at 3500 rpm for 10 min (Megafure 1.0 R Heraeus; DJB Lab Care, Germany). Free thyroxine (T_4_), free triiodothyronine (T_3_), thyroid-stimulating hormone (TSH), cortisol, testosterone, estradiol were analyzed from serum by Immulite 2000 XPi immunoassay system (Siemens Healthineers, Erlangen, Germany). Serum leptin was analyzed by Dynex Ds 2 ELISA prosessing System (DYNEX Technologies, Chantilly, VA, USA) using a commercial kit (Human leptin ELISA, BioVendor, Heidelberg, Germany). These hormones are routinely analyzed in our laboratory and day-to day reliability (CV%) for all of these hormones in our laboratory is <8%.

### Profile of Moods (POMS) and reproductive function questionnaires

Mood and menstrual bleeding was asked by the questionnaires at pre time-point, in the middle of the pre and mid timepoints and the week before the mid- and post measurements. The participant's mood was examined using the Finnish version (Vuoskoski and Eerola, 2011) of the Profile of Mood States-Adolescents questionnaire (POMS-A; Terry et al., 1999). The 24 items of the POMS, designed to measure six moods of Vigor, Confusion, Anger, Fatigue, Depression, and Tension, were rated on a 5-point Likert scale ranging from 1 = not at all to 5 = extremely.

Menstrual/reproductive function questionnaire involves yes or no answers to the following questions: Have you had some of these menstrual irregularities within the last 2 months: 1: menstrual cycle has become irregular, 2: menstrual bleeding has decreased, 3: menstrual bleeding has increased, and 4: no menstrual bleeding.

### Statistical analysis

All data are expressed as means ± SD, except where designated. Normality, skewness and possible outliers were checked with the Shapiro–Wilk test and several plots. Data was analyzed with the repeated ANOVA (time × group) with contrasts. Logarithm transformations were applied when needed to normalize variables or to homogenize group variances. If assumptions for parametric tests were not met even with transformations, nonparametric tests (Friedman's test, Sign test and Mann–Whitney test) were utilized, and variables were RANK-transformed for the estimation of interaction term (time × group in parametric ANOVA). Average percentage changes between groups were tested with the nonparametric Mann–Whitney test due to deviations from normality and outliers. Holm–Bonferroni was used to manually correct for the multi-tests between individual time-points between the groups and within the groups. IBM SPSS for Windows 22.0 (Armonk NY, 2013) was utilized for statistical analyses. Significance level was set as 0.05.

## Results

### Nutrition

The energy intake during the diet was on average 22.9 ± 13.8% (*p* < 0.001) lower than before the diet, while the energy intake of the control group remained unaltered (Table 1). The decreased energy intake in the diet group was mainly explained by reduced carbohydrate ingestion. Although, absolute protein and fat intake slightly decreased, these values were explained by decreased weight and, thus, per kg of body mass only very slight decrease in fat and no changes in protein intake was noticed (Table 1). Actually, the relative protein intake (% of total energy) increased (*p* < 0.001) and relative CHO intake decreased (*p* < 0.01), while relative fat intake remained unaltered (Supplementary Table 1). During the recovery energy intake of the participants returned to baseline levels.

**Table 1:** **Macronutrient consumption**.

	**Pre**	**Mid-diet**	**Diet average**	**Competition-week**	**Recovery**	**Group × time (p)**
**ENERGY (kJ)**						
Diet	9903.7 ± 1785.8	7887.6 ± 1440.9^***^	7524.2 ± 1556.2^***^	9789.7 ± 2553.8	9273.4 ± 2186.6	< 0.01
Cont	10446.1 ± 2307.5			9795.0 ± 1774.0	10425.5 ± 1549.8	
**ENERGY (kJ/kg bw)**						
Diet	155.0 ± 27.8	131.4 ± 24.4^***^	125.4 ± 26.2^***^	166.0 ± 61.1	158.8 ± 41.4	0.067
Cont	162.6 ± 34.7			151.9 ± 24.7	163.6 ± 24.5	
**PROTEINS (g)**						
Diet	202.5 ± 44.1	189.7 ± 39.5^*^	184.7 ± 40.5^*^	160.6 ± 32.5^**^	195.4 ± 41.5	< 0.05
Cont	172.3 ± 37.6			181.9 ± 37.9	184.7 ± 39.5	
**PROTEINS (g/kg bw)**						
Diet	3.16 ± 0.61	3.14 ± 0.63	3.07 ± 0.64	2.84 ± 0.52	3.34 ± 0.81	0.282
Cont	2.68 ± 0.53			2.81 ± 0.48	2.87 ± 0.85	
**CHO (g)**						
Diet	215.6 ± 67.7	126.1 ± 49.1^***^	127.8 ± 39.7^***^	229.9 ± 199.0	188.5 ± 72.5	< 0.01
Cont	218.8 ± 50.3			216.7 ± 41.4	224.0 ± 49.8	
**CHO (g/kg bw)**						
Diet	3.35 ± 0.99	2.10 ± 0.84^***^	2.12 ± 0.66^***^	4.10 ± 1.65	3.24 ± 1.34	< 0.01
Cont	3.40 ± 0.72			3.37 ± 0.63	3.52 ± 1.05	
**FAT (g)**						
Diet	64.4 ± 16.2	56.8 ± 16.4^*^	52.8 ± 16.4^***^	63.9 ± 25.3	59.7 ± 13.0	0.184
Cont	82.2 ± 23.3			73.8 ± 28.8	84.9 ± 29.4	
**FAT (g/kg bw)**						
Diet	1.02 ± 0.29	0.95 ± 0.29	0.88 ± 0.29^*^	1.07 ± 0.49	1.02 ± 0.23	0.692
Cont	1.29 ± 0.38			1.14 ± 0.44	1.34 ± 0.51	

The results are averaged daily values. ^*^, ^**^, and ^***^ p < 0.05–0.001 change vs. pre. bw, body weight; CHO, carbohydrates. Diet × group interaction includes pre and diet average time-points. Data is n = 27 diet and n = 18 control participants at pre and mid/average, n = 21 for the competition week and n = 18 and n = 16 for the diet- and control participants at the recovery. Recovery nutrition diaries were collected from the middle of the recovery period.

### Exercise

The training frequencies and MET-hours of the participants are shown in Table 2. The participants resistance trained 4.7 ± 0.7 (diet group) and 3.9 ± 1.9 (controls) times per week at the time of the pre-measurements. The diet group maintained their resistance training frequency during the diet, during the competition week and during the recovery period. However, when taking into account the reported length and intensity of their resistance exercise bouts, the MET-hours of the participants decreased during the competition week (*p* < 0.001), probably due to the tapering period fitness-competitors have before the competition. The controls maintained their resistance training frequencies and MET-hours throughout the study period. Lower body muscles were trained during the diet 1.4 ± 0.5 times per week and specific upper body muscle groups 1.1 ± 0.3 times per week in a split design. Unlike resistance training, the diet group increased their aerobic training during the diet (4.9 ± 2.9 times per week) when compared to pre-state (3.6 ± 2.8; *P* < 0.05 and diet × group interaction *p* = 0.013). This was due to the increased amount of steady state aerobics in several subjects, while part of the participants also started to conduct or increased the amount of HIT-exercises. The level of aerobic training decreased during the recovery period in these participants down to 2.3 ± 1.9 times per week while the controls maintained their levels of aerobic training.

**Table 2:** **Exercise levels**.

	**Pre**	**Mid-diet**	**Second-last week**	**Competition-week**	**Recovery**	**Group × time (p)**
**RE x/wk**						
Diet	4.7 ± 0.7	4.7 ± 0.6	4.7 ± 0.7	4.2 ± 1.1	4.6 ± 0.8	0.961
Cont	3.9 ± 1.9		3.8 ± 1.7		3.8 ± 1.7	
**RE METh/wk**						
Diet	44.9 ± 8.6	46.0 ± 8.5	45.6 ± 9.7	19.5 ± 10.9^***^	42.4 ± 8.3	0.186
Cont	31.9 ± 19.5		27.8 ± 14.9		32.2 ± 17.2	
**AE x/wk**						
Diet	3.6 ± 2.8	4.4 ± 2.9	4.9 ± 2.9^*^	4.4 ± 2.5	2.3 ± 1.9##	0.013
Cont	2.6 ± 2.6		2.9 ± 2.4		3.3 ± 3.5	
**AE METh/wk**						
Diet	13.3 ± 10.4	19.1 ± 15.6	22.0 ± 17.1	13.2 ± 8.4	9.7 ± 7.8#	0.015
Cont	17.9 ± 23.7		14.9 ± 14.9		18.3 ± 24.8	

^*^, ^**^, and ^***^ is significant p < 0.05–0.001 change vs. pre and #–## is significant (p < 0.05–< 0.01) difference between the groups in the change. RE, resistance exercise; AE, aerobic exercise. Of the RE-results, n = 24–26 for the diet participants and n = 18 for the controls. Regarding the AE, n = 21–26 and n = 19 for the diet and control participants, respectively.

### Body composition

**Figure 2:**
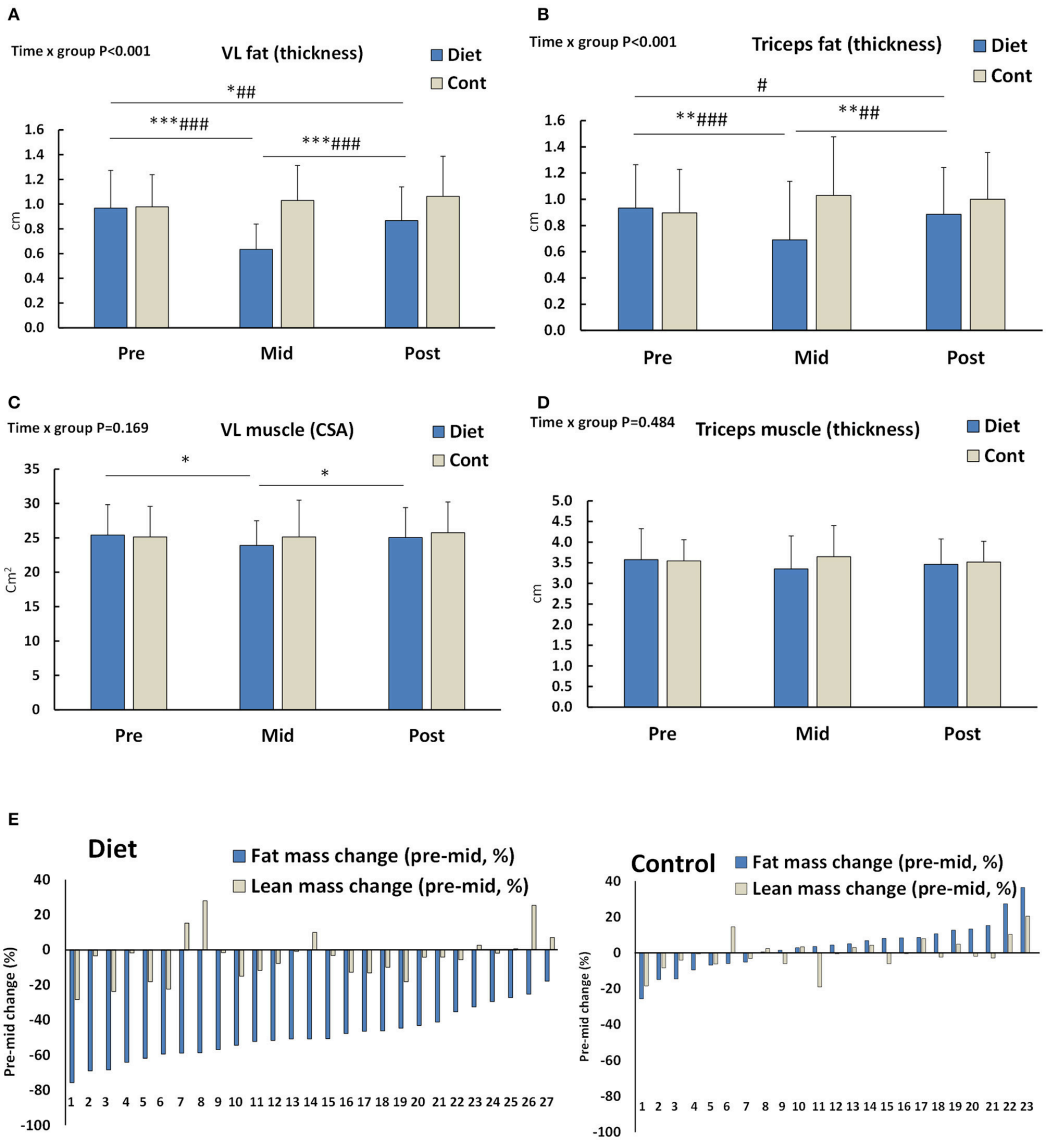
**(A,B)** Subcutaneous fat thickness around Vastus lateralis (VL) and triceps brachii muscles (*n* = 25–27 diet and 19–20 control participants). **(C,D)** VL muscle cross-sectional area (CSA) and triceps brachii muscle thickness (*n* = 27 diet and 20 control participants). **(E)** Individual fat and lean mass percentage changes during the diet or pre to mid control period. Number at the x-axis depicts participant numbers ordered based on the amount of fat loss. DXA and bioimpedance values are averaged (*n* = 27 diet and 23 control participants). ^*^–^***^ is significant (*p* < 0.05–< 0.001) difference to Pre and #–### is significant (*p* < 0.05–< 0.001) difference between the groups in the change. Group × time = ANOVA interaction effect *p*-value.

Body composition was examined with many indirect measurements [DXA, bioimpedance (InBody) and skinfolds] as well as direct measures (ultrasound) as the diet itself may distort some of the assumptions of individual body composition measurements or their formulas used. There was group × time interaction (*p* < 0.001) in body mass and fat mass independent of the method used (DXA, skinfolds, bioimpedance; Table 3) as well as in fat thickness (Figures 2A,B; ultrasound). These were explained by the fact that the body and fat mass of the diet group participants decreased during the diet and returned almost completely (body mass) or partially (fat mass and thickness) to the baseline after the recovery period. No change of these parameters was observed in the control group (Table 3). This resulted in a decrease in fat % by diet on average from 23.1 ± 5.6 to 12.7 ± 4.0% (DXA), from 19.7 ± 4.2 to 11.6 ± 3.9% (bioimpedance), and from 25.2 ± 3.0 to 18.3 ± 2.7% (skinfolds). From these values, fat % increased during the recovery period to 20.1 ± 5.4%, 18.0 ± 4.5%, and 25.9 ± 4.1% based on DXA, bioimpedance, and skinfold measurements, respectively. Regarding individual areas, the android region in DXA reflecting visceral fat decreased ~68% in the diet group (*p* < 0.001) and then recovered toward baseline and controls (Table 3). Similarly, fat in the gynoid area decreased by diet 44.2 ± 12.8% (*p* < 0.001) followed by an increase close to the pre-values during the recovery period (post–pre: −9.4 ± 19.5%, *p* < 0.05) while remaining unaltered in the controls (diet × group interaction *p* < 0.001).

**Table 3:** **Body composition of the subjects**.

	**Pre**	**Mid**	**Post**	**ΔMid-Pre**	**ΔPost-Mid**	**ΔPost-Pre**	**Group × time (p)**
**HEIGHT (cm)**							
Diet	165.3 ± 4.3						
Cont	165.9 ± 5.7						
**BODY MASS (kg)**							
Diet	64.3 ± 6.9	56.5 ± 5.3	62.6 ± 6.9	−11.9 ± 3.8%^***^	10.9 ± 5.3%^***^	−2.4 ± 5.6%^*^	< 0.001
Cont	64.3 ± 5.3	64.5 ± 5.5	64.4 ± 5.5	0.3 ± 3.1%###	−0.1 ± 3.4###	0.1 ± 3.7%	
**FAT MASS (DXA: kg)**							
Diet	14.6 ± 4.6	7.1 ± 2.7	12.7 ± 4.2	−50.4 ± 12.4%^***^	84.7 ± 50.7%^***^	−10.1 ± 25.6%^*^	< 0.001
Cont	14.9 ± 3.7	15.5 ± 3.9	15.1 ± 4.4	5.1 ± 12.9%###	−2.6 ± 13.2%###	2.1 ± 16.8%	
**FAT MASS (InBody: kg)**							
Diet	12.8 ± 3.4	6.6 ± 2.5	11.2 ± 3.3	−47.4 ± 17.9%^***^	91.5 ± 74.0%^***^	−6.1 ± 29.3%	< 0.001
Cont	13.0 ± 3.1	13.2 ± 3.5	13.0 ± 4.0	2.1 ± 16.2%###	−1.5 ± 15.6%###	−0.1 ± 17.8%	
**FAT MASS (SKINFOLDS: kg)**							
Diet	16.3 ± 3.3	10.4 ± 2.2	16.4 ± 4.0	−35.2 ± 11.2%#^***^	57.9 ± 26.7%^***^	1.3 ± 18.8%	< 0.001
Cont	15.5 ± 9.4	16.2 ± 9.7	16.3 ± 9.9	9.5 ± 29.6%	−4.5 ± 43.6%	9.5 ± 52.4%	
**ANDROID FAT (DXA: kg)**							
Diet	0.92 ± 0.34	0.25 ± 0.14	0.82 ± 0.30	−68.1 ± 19.8%^***^	276 ± 204.2%^***^	2.4 ± 53.6%	< 0.001
Cont	0.98 ± 0.38	1.05 ± 0.43	0.98 ± 0.48	7.8 ± 17.7%###	−6.2 ± 23.4%###	1.2 ± 29.2%	
**LEAN MASS (DXA: kg)**							
Diet	47.6 ± 4.1	48.0 ± 3.9	48.3 ± 4.3	1.1 ± 3.1%	0.6 ± 3.8%	1.7 ± 4.4%	0.272
Cont	47.3 ± 4.7	47.2 ± 4.7	47.4 ± 5.0	−0.2 ± 2.4%	0.5 ± 2.7%	0.3 ± 3.1%	
**LEAN MASS (InBody: kg)**							
Diet	48.6 ± 4.9	47.1 ± 4.3	47.9 ± 5.0	−3.0 ± 4.4%^**^	2.1 ± 3.2%^**^	−1.1 ± 4.7%	0.341
Cont	49.2 ± 4.9	48.4 ± 4.8	48.5 ± 4.6	−1.6 ± 5.8%	0.2 ± 2.9%	−1.3 ± 6.1%	
**FFM (SKINFOLDS: kg)**							
Diet	48.0 ± 4.3	46.1 ± 3.9	46.3 ± 4.1	−3.9 ± 2.7%#^***^	0.4 ± 2.4%	−3.5 ± 3.1%^***^	< 0.05
Cont	48.8 ± 6.8	48.2 ± 7.0	48.0 ± 7.1	−1.2 ± 3.1%	−0.4 ± 4.0%	−1.6 ± 4.5%	
**TOTAL BONE (DXA: kg)**							
Diet	2.56 ± 0.28	2.53 ± 0.29	2.56 ± 0.31	−1.3 ± 1.8%^***^	1.5 ± 3.6%	0.1 ± 3.6%	0.104
Cont	2.57 ± 0.28	2.58 ± 0.29	2.61 ± 0.29	0.3 ± 1.3%##	1.3 ± 2.7%	1.6 ± 2.5%#^*^	

Δ are %-changes. ^*^–^***^ is significant (p < 0.05–< 0.001) difference to Pre and #–### is significant (p < 0.05–< 0.001) difference between the groups in the change. Group × time = ANOVA interaction effect p-value. n = 27 diet and 23 control participants for the variables except InBody in which n = 25 for diet subjects for the post-measurement. FFM = fat-free mass.

Unlike body mass and fat mass, diet or the recovery period did not significantly alter lean mass measured by DXA. There was, however, a small, but statistically significant decrease in lean mass, fat-free mass (Table 3), and VL-muscle CSA (Figure 2C) measured by bioimpedance, skinfolds, and ultrasonography, respectively. These small changes, when existing, were fully recovered during the recovery period (Table 3, Figure 2C). No significant change was observed in the triceps brachii muscle thickness due to diet or the recovery period (Figure 2D). When individual values were looked at more closely, all the females had medium to large decrease in fat mass after diet, but some females of the diet group had a small increase in their lean mass (average of DXA and bioimpedance) while most of them had a decrease or no change (Figure 2E).

Total bone mass tended to have group × time interaction (*p* = 0.10), which was explained by a decrease in bone mass during diet (*p* < 0.001) while no change was observed in the controls (*p* > 0.3; Table 3).

### Maximal and explosive strength

Isometric maximal strength and explosive strength of leg extensors remained unchanged during diet and there was no difference compared to controls (group × time interaction *p* > 0.1; Table 4). However, isometric bench press decreased during the diet when compared to control participants (*p* < 0.05; Table 4).

**Table 4:** **Muscle force of the subjects**.

	**Pre**	**Mid**	**Post**	**ΔMid-Pre**	**ΔPost-Mid**	**ΔPost-Pre**	**Group × time (p)**
**LEG PRESS (N)**							
Diet	2855 ± 430	2855 ± 656	3059 ± 538^**^	−0.5 ± 9.5%	8.9 ± 12.2%	6.8 ± 9.7%	0.316
Cont	2908 ± 315	2893 ± 388	3021 ± 454	−0.5 ± 6.3%	4.2 ± 8.1%	2.5 ± 8.1%	
**BENCH PRESS (N)**							
Diet	620.0 ± 104.1	581.1 ± 104.4	598.9 ± 104.4	−3.4 ± 7.5%#	−0.2 ± 8.0%###	−3.7 ± 8.2%	< 0.001
Cont	645.6 ± 91.4	662.6 ± 82.3	602.8 ± 84.5^***^	2.1 ± 5.8%	−8.7 ± 3.8%	−6.9 ± 5.4%	
**VERTICAL JUMP (cm)**							
Diet	26.1 ± 4.2	25.2 ± 4.5	26.3 ± 4.7	−3.4 ± 9.1%	4.7 ± 8.9%	0.9 ± 9.6%	0.107
Cont	28.1 ± 5.5	28.4 ± 5.0	28.2 ± 5.1	1.6 ± 10.4%	−0.4 ± 7.7%	0.8 ± 9.2%	

Δ are %-changes. ^*^–^***^ is significant (p < 0.05–< 0.001) difference to Pre and #–### is significant (p < 0.05–< 0.001) difference between the groups in the change. Group × time = ANOVA interaction effect p-value. n = 27 diet and 23 control participants for the vertical jump and for isometric leg press and bench press n = 25 and 26 for the diet participants and n = 20 and n = 23 for the controls, respectively.

### Hormones

**Figure 3:**
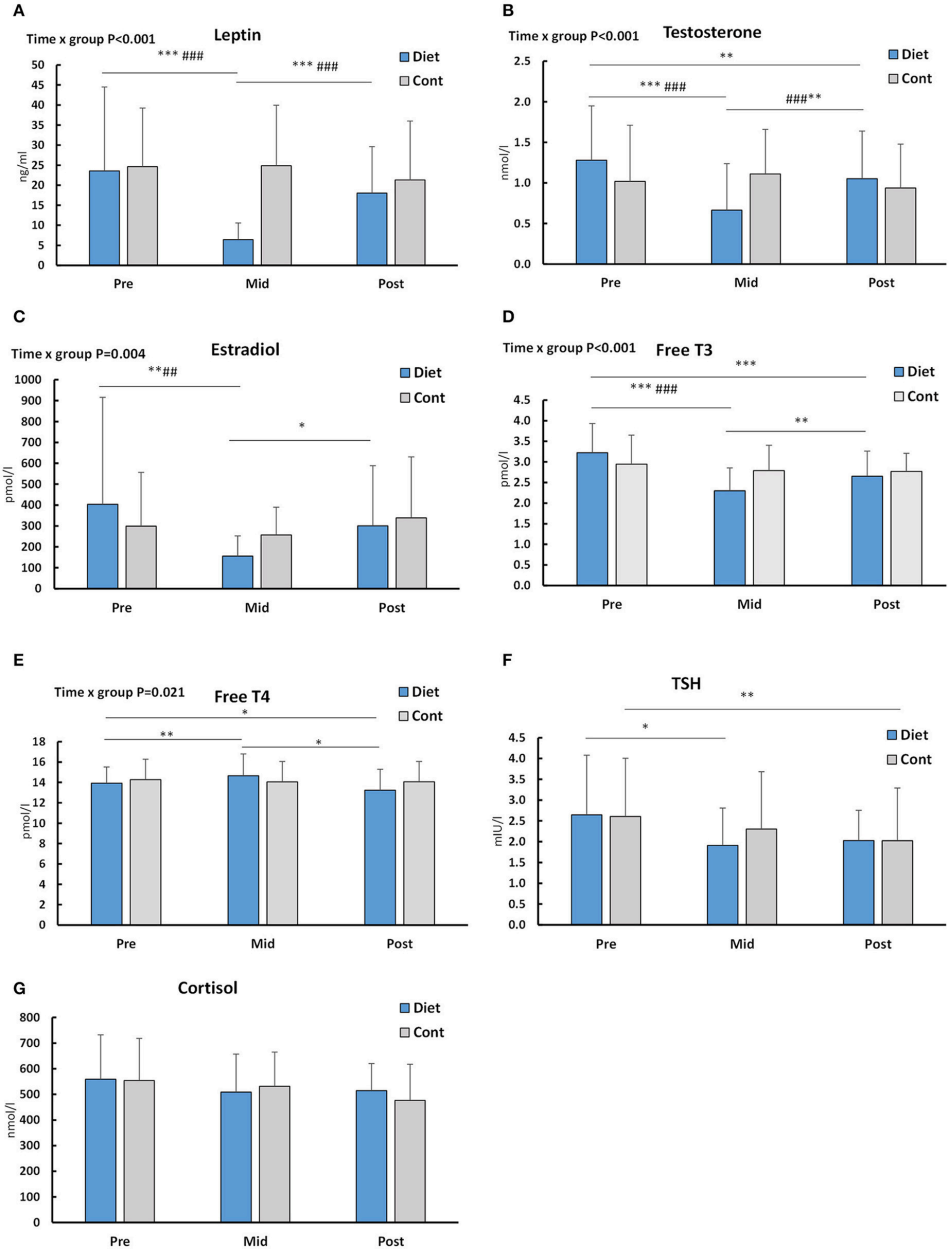
**Serum hormone concentrations at baseline (Pre), after the diet/control period (Mid) and after that during the recovery period (Post)**. Out of individual panels, **(A)** depicts leptin, **(B)** testosterone, **(C)** estradiol, **(D)** free T_3_, **(E)** free T_4_, **(F)** TSH, and **(G)** cortisol. *n* = 27 diet and 23 control participants for all hormones except TSH and testosterone data, which is obtained from 22 controls. ^*^–^***^ is significant (*p* < 0.05–< 0.001) difference to Pre and #–### is significant (*p* < 0.05–< 0.001) difference between the groups in the change. Significant (*p* < 0.05) group × time = ANOVA interaction effect *p*-values are shown. For TSH and cortisol there were no significant interaction effects (*p* = 0.198 and *p* = 0.332, respectively).

**Figure 4:**
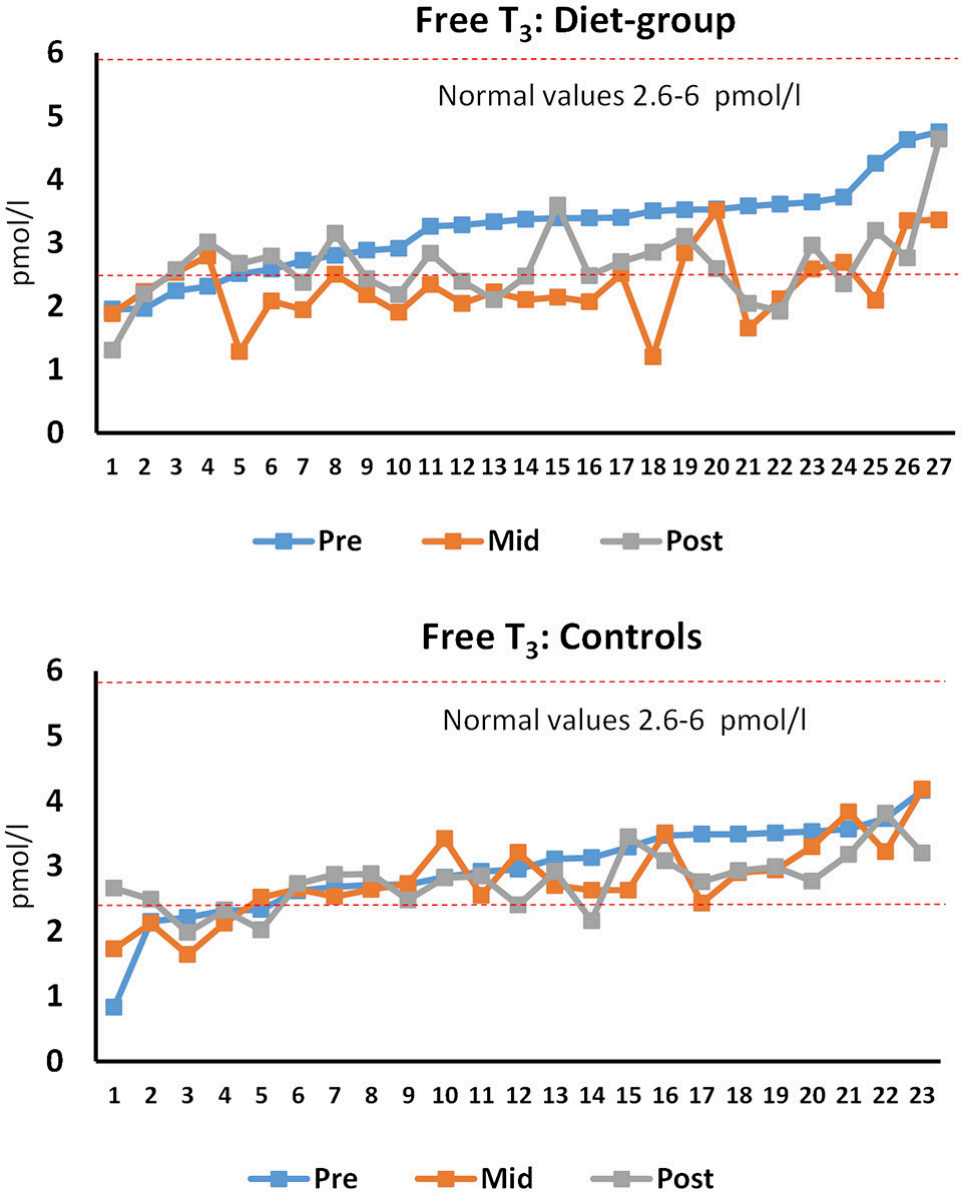
**Individual free T_**3**_ at pre, mid, and post time-points for diet and control participants**. Number at the x-axis depicts participant numbers ordered based on the pre-value. The red dotted lines limit the reference values in normal-weight healthy females. *n* = 27 diet and 23 control participants.

There was group × time interaction in serum concentrations of leptin, testosterone, T_3_ (*p* < 0.001), and estradiol (*p* < 0.01; Figures 3A–D). This was shown as decreases in these hormones due to the diet when compared to the control group. Leptin and estradiol (Figures 3A,C) increased back to baseline while T_3_ (Figures 3D, 4) and testosterone (Figure 3B) remained slightly, but significantly below the baseline even after the recovery period. No changes to the controls at the post timepoints were, however observed. T_4_ showed an increase due to diet and a reduction back to baseline during the recovery period, but these changes were small and non-significant in comparison to controls (Figure 3E). No treatment × time interaction was observed in TSH and cortisol (*p* > 0.19, Figures 3F,G).

### Blood pressure, heart rate, hemoglobin, and hematocrit

Systolic blood pressure showed a group × time interaction (*p* < 0.001), whereas no effect was observed on diastolic blood pressure (*p* = 0.177) (Table 5). The *post-hoc* analysis showed that diet led to decreased systolic blood pressure (*p* < 0.001), which remained decreased after the recovery period, when compared to baseline (*p* < 0.01) and to the controls (*p* < 0.05). Similarly, heart rate showed a group × time interaction (*p* < 0.007) with a decrease-effect found with the diet (*p* < 0.001) that was sustained still after the recovery period (*p* < 0.01; Table 5). Blood hemoglobin showed a group × time interaction (*p* < 0.017), while a trend existed with hematocrit (*p* = 0.076; Table 5). The *post-hoc* analysis showed that diet decreased hemoglobin (*p* < 0.01), but an increase back to baseline was observed after the recovery period.

**Table 5:** **Blood pressure and hemoglobin/hematocrit of the subjects**.

	**Pre**	**Mid**	**Post**	**ΔMid-Pre**	**ΔPost-Mid**	**ΔPost-Pre**	**Group × time (p)**
**SYSTOLIC BLOOD PRESSURE (mmHg)**							
Diet	118.3 ± 9.3	111.1 ± 7.6	113.9 ± 7.2	−5.9 ± 5.9%^***^	2.6 ± 5.3%^*^	−3.5 ± 5.4%^**^	< 0.001
Cont	119.3 ± 6.3	121.7 ± 7.7	119.2 ± 11.0	2.0 ± 2.1%^***^###	−2.1 ± 7.3%#	−0.1 ± 7.5%#	
**DIASTOLIC BLOOD PRESSURE (mmHg)**							
Diet	67.4 ± 8.0	64.1 ± 6.4	65.3 ± 6.4	−4.0 ± 10.2%	2.1 ± 8.3%	−2.5 ± 8.1%	0.177
Cont	69.1 ± 7.1	70.1 ± 13.3	67.7 ± 7.4	1.7 ± 18.1%	−1.0 ± 16.1%	−1.9 ± 5.6%	
**HEART RATE (bpm)**							
Diet	68.9 ± 12.1	62.4 ± 9.0	64.6 ± 10.1	−8.4 ± 10.6%^***^	4.0 ± 11.5%	−5.5 ± 8.9%^**^	0.007
Cont	69.6 ± 8.5	70.1 ± 13.3	68.8 ± 9.8	0.2 ± 10.5%#	−0.4 ± 12.3%	−1.1 ± 8.1%	
**HEMOGLOBIN (g/L)**							
Diet	138.8 ± 8.1	134.7 ± 7.8	137.7 ± 7.7	−2.9 ± 4.1%^**^	2.4 ± 4.3%^*^	−0.7 ± 3.4%	0.017
Cont	135.5 ± 6.1	135.3 ± 5.7	134.9 ± 7.2	0.0 ± 3.6%	−0.3 ± 3.6%	−0.4 ± 4.0%	
**HEMATOCRIT (%)**							
Diet	40.8 ± 2.3	39.8 ± 2.4	40.8 ± 2.2	−2.3 ± 4.4%	2.6 ± 4.4%	0.2 ± 3.6	0.076
Cont	40.3 ± 1.5	40.0 ± 1.6	40.1 ± 1.8	−0.6 ± 3.0%	0.1 ± 3.5%	−0.5 ± 3.8%	

Δ are %-changes. ^*^–^***^ is significant (p < 0.05–< 0.001) difference to Pre and #–### is significant (p < 0.05–< 0.001) difference between the groups in the change. Group × time = ANOVA interaction effect p-value. n = 27 diet and 23 control participants for all the result.

### Menstrual irregularities

The baseline values for the missing menses in the diet-participants was 11.1% (3/27) and for irregularities in menstrual bleeding 37.0% (10/27) while in the controls the same values were and 4.3% (1/23) and 30.4% (7/23) without differences between the groups (*p* > 0.6). Fisher's exact test revealed that the diet group had more (63%) irregular menstrual bleeding than controls (30%; *p* < 0.05) and tended to have more missing menses (44 vs. 22%, *p* = 0.082) during the diet than the controls during their weight-maintenance period (measurement-points in the middle of the diet or after the diet before the competition). After the recovery period at post, in the diet group 28% (7/25) females had no menstrual bleeding and in the controls the respective value was 14.0% (3/22; *p* = 0.297). For those competitors using estrogen containing contraception (*n* = 19) there were irregularities in menstrual bleeding during the diet in 10/19 participants and/or lack of menstruation in 8/19 participants (at baseline 8/19 and 2/19, respectively). On the other hand, those subjects not using estrogen containing contraception (*n* = 5), 5/5 demonstrated irregular menstrual bleeding during the diet and/or lack of menstruation in 3/5 participants (at baseline 2/5 and 1/5, respectively). Three subjects did not report whether they used contraception medication or not.

### Mood

There was no statistically significant changes in the mood of the participants within the diet group or between the diet and control groups at any of the time-points (Supplementary Table 2). The only statistical trend was a slight decrease in the vigor of the competitors (*p* = 0.066) in the middle of the diet when compared to the changes in the control group participants.

## Discussion

The present study showed that a diet to achieve very low levels of body fat can be completed with very small losses of lean mass/muscle size and muscle function in normal weight females with high levels of protein intake and resistance exercise. Moreover, the endocrine system is altered during the diet, but is recovered in most of the females following a recovery period of 3–4 months that includes increased energy intake together with the recovery of body weight.

Previous studies with energy deficits in otherwise normal weight individuals have shown various results in body composition. In the classic Minnesota starvation study body mass decreased in males on average by 25% with lean mass representing 6–28% (mean ~15%), and the rest being fat (Keys et al., 1950; Dulloo et al., 1996). On the other hand, fat-free mass showed only a small decrease during a diet in female bodybuilders (van der Ploeg et al., 2001). There are a few possible explanations why lean mass and muscle size were maintained in the present study. First, the proportion of lean mass loss during diet has been larger the smaller the initial body fat% was (Dulloo et al., 1996; Huovinen et al., 2015). In the present study, females had an average 19–25% of fat (depending on whether DXA, bioimpedance, or skinfolds was used) at baseline, which is relatively high in comparison to e.g., ~14% of fat in the male participants of the Minnesota study (Keys et al., 1950; Dulloo et al., 1996). Second, studies have shown that during energy deficit lean mass can be better maintained with ≥1.7–2 g/kg body weight per day protein ingestion in males and females (Mettler et al., 2010; Josse et al., 2011; Arciero et al., 2013; Churchward-Venne et al., 2013; Pasiakos et al., 2013; Longland et al., 2016). In our study cohort of fitness-competitors the protein content remained high, on average 3 g/kg even during the energy deficit. Third, resistance and aerobic training have been shown to attenuate the loss of lean mass during energy deficit when compared to energy restriction alone (Kraemer et al., 1999; Miller et al., 2013), which were both also used during the diet by the present fitness competitors. Moreover, the changes in body weight of the diet group were rather slow, on average ~0.4 g/kg per week, which is actually pretty close to the level that was previously observed to maintain lean mass in normal-weight females on a diet (Mero et al., 2010) and what has been recommended for fat loss in fitness diets (Helms et al., 2014). The average participant, while resistance trained, had not had many years of training experience (average 3.5 ± 1.4 years in the diet group and 3.1 ± 1.2 years in the controls), which makes it possible for some to even gain some muscle mass on a diet when regularly conducting resistance training combined with high protein intake (Josse et al., 2011; Longland et al., 2016). It is also important to recognize that the last week of a typical fitness or bodybuilding diet includes tapering of training, which was observed as decreased MET-hours of resistance training and similar to baseline carbohydrate eating. This makes lean mass and muscle sizes after diet more comparable when compared to baseline. When this is not taken into account in diet studies in which carbohydrates are restricted, small 1–3 kg losses in lean mass may just be glycogen and water losses instead of an actual decrease in protein mass.

Out of the present serum hormones investigated the concentration of leptin robustly decreased but recovered close to baseline levels after the recovery period following energy intake and fat mass as expected (Kelesidis et al., 2010). Decreased leptin after diet has orexigenic effects in response to energy deficiency and in decreased fat mass (Kelesidis et al., 2010) and may be one of the reasons why maintaining low energy intake and energy deficit for a long time has been shown to be psychologically very difficult (Keys et al., 1950). In our study, only a few of the females had leptin levels <3 ng/ml (6 of 27 diet-participants) after the diet, a level thought to be a low-limit for many physiological changes in females such as decreased immune function (Chan et al., 2006). This state was reversed by the recovery period as serum leptin levels did recover to ≥3 ng/ml levels in all females. This is important as recovery of leptin can in itself restore, for instance, ovulatory menstrual cycles and improve levels of reproductive and thyroid hormones as well as bone markers in amenorrhea (Rosenbaum et al., 2002; Kelesidis et al., 2010).

Weight reduction in obese or overweight individuals typically decreases T_3_ while T_4_ and TSH usually remain unaltered (Fothergill et al., 2016). We now showed that also in normal weight to thin individuals, weight loss from mainly fat stores decreases only T_3_ and not T_4_ and TSH. This is also supported by data from individuals with anorexia nervosa who have lowered serum leptin and T_3_, together with low fat mass (Tolle et al., 2003). These hormones are usually at least partially recovered toward the values of normal-weight individuals after weight gain (Tolle et al., 2003). Individual T_3_ levels were also compared to the reference values. For T_3_, in dieters, 5/27 were below the reference values (2.6–6 pmol/L) at pre, 20/27 after the diet and 13/27 at recovery while in controls the number of participants was 5/23, 8/23, and 7/23, respectively (Figure 3). Statistically significantly lowered T_3_ at the post time-point suggests that 3–4 months of recovery may not be enough in some individuals after heavy energy deficit. These females should probably continue the recovery period of increased energy intake before dieting again for the competition to avoid longer term changes in their hormonal balance. This is because in addition to possible health risks, decreased T_3_ can lead to decreased metabolic rate (Kim, 2008). The decrease and only a partial recovery of T_3_ was accompanied by the decreased heart rate and systolic blood pressure. Similarly as T_3_, heart rate also remained decreased after the recovery period. Together with decreased fat mass, decreased blood pressure and heart rate are typical responses after a diet, even for normotensive and non-overweight individuals (Keys et al., 1950; Awazu et al., 2000; Rossow et al., 2013) showing that the autonomic nervous system also adapts to an energy deficit as expected and, thus, may not be a health risk at these levels of heart rate of ≥49 bpm in all females. However, when the heart rate is more substantially decreased and long lasting such as in anorectic individuals without supervised and controlled refeeding, this can lead to a long QT in ECG and an increased risk for arrhytmia (Sachs et al., 2016).

Anorectic females have very low serum estradiol (Tolle et al., 2003) and testosterone (Miller et al., 2007). Not surprisingly, in the present study, serum sex-hormones estradiol and testosterone both decreased during the diet. In addition, estradiol, but not testosterone, recovered at or close to baseline levels within 3–4 months of recovery. This suggests that recovery of serum testosterone in females takes longer time and/or larger energy intake and/or fat mass when compared to estradiol. It is unknown whether the levels observed in the present study are physiologically meaningful as the mean values are within normal values of serum testosterone in most of the participants (Haring et al., 2012) and the measured loss of muscle size was very small. However, higher serum testosterone levels in females have been associated with larger lean mass to fat mass ratio (Rickenlund et al., 2003) and occasionally correlation between gains in muscle cross-sectional area and/or strength during resistance training have also been observed (Häkkinen et al., 1992). Interestingly, a slightly decreased bone mass by DXA was observed in diet, increasing back to baseline after the recovery period. The result may be due to the direct effects of decreased body and muscle mass on mechanical forces (Goodman et al., 2015), decreased energy availability (Ihle and Loucks, 2004) and/or lowered estradiol and testosterone that are both anabolic to bone based on some (Davis et al., 1995), but not all studies (Muñoz et al., 2002).

Some studies have suggested that for optimal health, minimum body fat for females would be within the range of 12–14% (Meyer et al., 2013), but this depends on the individual and the body composition assessment method. BMI decreased from 23.5 ± 1.8 to 20.6 ± 1.4 recovering to 22.9 ± 2.0 while the lowest BMI's after the diet were 18.7–19.0 in four females. On the other hand, 6/27 females had <10% body fat after the diet in both DXA and in bioimpedance. In many sports such as in distance running, figure skating and gymnastics fat % in females can be as low as 10–15% and in some females even below 10% almost year round (Wilmore et al., 1977). Instead, in fitness-competitors, fat percentage is usually kept at low levels only for a rather short period of time. Indeed, none of the participants had <10% levels after the recovery period, while levels of 15–20% were frequent. This may, in theory, offer a health protection. Weight cycling, i.e., repeated cycles of weight loss and regain that are observed in those who frequently compete and thus diet may, however, predispose to some health risks such as obesity in some groups of people (Saarni et al., 2006), although the total weight of evidence is very weak (Mehta et al., 2014). Nevertheless, females need a higher fat percentage than males because of the endocrine function that is necessary for ovulation and maintenance of bone mass. Indeed, in the present study, more dieting females reported changes in their menstrual function during their diet than controls in their control period. The values reported are not unexpected as menstrual dysfunction is very common among many thin female athletes (Melin et al., 2015). These results are probably related, in part, to changes in fat mass around visceral and genital areas. In the present study the estimation of visceral fat measured as an android region mass by DXA showed a substantial ~68% decrease followed by an increase back to the pre-values after the recovery period. A recent meta-analysis suggests that in negative energy balance, both exercise and diet reduce fat mass, but exercise may be more important to reduce visceral adipose tissue (Verheggen et al., 2016). The decrease in fat in these areas combined with energy deficit can, in theory, signal the hypothalamus-pituitary axis to decrease secretion of hormones relating to ovulation such as estradiol and eventually, to transiently stop ovulation and menstrual bleeding.

Even though muscle strength is not essential for fitness competition performance *per se*, it may be, in theory, important to maintain muscle strength for the purposes of resistance training adaptations. In the present study, the participants maintained their maximal isometric muscle strength and explosive strength of leg extensors during the diet. This can be explained, in part by maintenance of muscle size in the important knee extensor muscles investigated and by frequent heavy intensity resistance training that was maintained throughout the study, which can sustain or improve voluntary activation of muscles even in strength trained female athletes (Häkkinen and Kallinen, 1994). However, a measure of upper-body strength, isometric bench press, decreased during diet when compared to controls suggesting that during the diet especially the upper-body strength may be difficult to maintain due to energy/carbohydrate deficit with a typical training program of fitness athletes. This was observed even though the thickness of triceps brachii muscle, which is one of the main muscles responsible for the bench press strength, remained unaltered during the diet. Previously, Bamman et al. (1993) noticed a decrease in isometric strength after a bodybuilding diet in males while Rossow et al. (2013) reported decreased absolute, but not relative levels of muscle strength and Robinson et al. (2015) reported no changes in most of the isokinetic measures of maximal strength in a male bodybuilder after the diet.

Mentally, maintaining rather a large energy deficit can be hard, possibly especially for those who are dieting toward very low body fat levels as observed previously in bodybuilders on a competition diet (Newton et al., 1993). Perhaps surprisingly, the present fitness-diet did not markedly affect the mood state of the participants. Supporting the mood results, serum cortisol did not increase following the diet. This was also a bit surprising considering that in stressful situations such as energy restriction and monitoring calories, serum cortisol level often increase (Tomiyama et al., 2010). However, a slight decrease in the vigor of the participants (*P* = 0.066) was observed in the middle of the diet when compared to the changes in the control group participants. This suggest that some individuals on the diet may have felt less active, lively, and/or energetic when compared to their baseline levels than individuals in the control group. This supports the earlier case-study with male drug-free bodybuilder (Rossow et al., 2013). The reason why the results were not very consistent may be explained, in part, by the fact that the participants were voluntarily taking part in the diet, which makes the situation different compared to a full randomized trial without a clear “reward” (competition) at the end.

The present study had several strengths. The n-size of the study was rather large and a control group was included, which is quite rare in energy deficit—refeeding studies. In addition, the comprehensive analysis of body composition with direct measures of muscle and fat size can be regarded as a strength. On the other hand, the study was limited in the experimental design that it was not randomized. However, we think that a similar background of the groups enables us to compare the changes in the diet group to almost an identical group of the controls not changing their nutrition or exercise during the experimental period. The physiology and body composition measurements were taken after a competition and not a week before the “peak” week. This is, however, in our view also an advantage as mentioned above at least regarding the body composition measurements. Contraceptive medication use did not result in exclusion from the study. In the present study the majority (19) of the competitors reported using estrogen-containing contraception and five not. While we acknowledge that in addition to the variation in the phase of the menstrual cycle, also oral contraception use can have effects and thus add extra variation on the measured variables. We analyzed the changes in serum estradiol from pre to mid and from mid to post and those remained significant in the estrogen containing contraception users and in non-users (*p* < 0.05) similarly as shown with all the subjects pooled (data not shown). Unfortunately the n-size is too small to conduct a valid statistical analysis for the menstrual irregularities, but our data tends to suggests that those who do not use estrogen containing medication may be slightly more vulnerable to menstrual irregularities during the diet than those who use them. However, this question needs to be addressed with studies using larger n-size in the future.

In conclusion, a fitness diet in healthy young females accomplished by restricting carbohydrate ingestion and increasing aerobic exercise while maintaining high levels of protein intake and resistance exercise can be carried out without major decreases in lean mass/muscle size. Therefore, the diet almost exclusively decreased body fat and altered serum hormones, but most of those values recovered within 3–4 months with the increase in energy intake and decrease in high level of aerobic exercise. However, in some females this time period may not have been long enough for a full recovery (e.g., free T_3_ and testosterone hormones). Future studies should investigate the time-course of the changes during the diet and in the recovery period and whether repeating heavy diets for many times has any long-lasting negative effects.

## Author contributions

JH, VI, JA, and KH conceived and designed the experiments with the help from MP and AW. VI, MS, and NJ carried out the experiments with the help from students and laboratory technicians. KN acted as a physician of the project. JH drafted the manuscript. Analysis was conducted by VI, MS, NJ, JH, and MK together with students and technicians. All authors critically read/revised and approved the final manuscript.

## Funding

This work was financially supported by the Academy of Finland (grant No. 275922 to JH and No. 269517 to MP), Finnish Fitness Sports Association and Department of Biology of Physical Activity.

### Conflict of interest statement

The authors declare that the research was conducted in the absence of any commercial or financial relationships that could be construed as a potential conflict of interest.
