# Supplementary material for: The Effects of Intensive Weight Reduction on Body Composition and Serum Hormones in Female Fitness Competitors
Source: Front Physiol. 2017 Jan 10;7:689. doi: 10.3389/fphys.2016.00689 (PMC5222856; doi:10.3389/fphys.2016.00689)
Supplement: Supplementary file 1 [file Table1.PDF]

Supplementary Table 1. Macronutrient consumption: % of energy

	Pre	Mid-diet	Diet average	Competition-week	Recovery	Group x time (p)
Proteins (g)						
Diet	32.3 ± 7.0	41.1 ± 6.7 ***	42.1 ± 6.5 ***	29.3 ± 8.1 *	37.0 ± 6.7	0.062
Cont	26.6 ± 6.3			31.9 ± 5.9	30.3 ± 6.2	
CHO (g)						
Diet	36.9 ± 7.1	26.7 ± 8.6 **	28.5 ± 5.8 **	41.6 ± 15.2	39.5 ± 8.6	<0.01
Cont	35.8 ± 5.3			37.7 ± 4.0	36.5 ± 6.0	
Fat (g)						
Diet	24.3 ± 4.7	27.0 ± 6.3	26.1 ± 5.8	24.0 ± 6.3	24.6 ± 4.1	0.156
Cont	30.0 ± 9.0			27.3 ± 7.1	29.9 ± 8.0	

*, ** and *** $p < 0.05\text{-}0.001$ change vs. pre. CHO = carbohydrates. Data is n=27 diet and n=18 control participants at pre and mid/average, n=21 for the competition week and n=18 and n=16 for the diet- and control participants at the recovery. Recovery nutrition diaries are from the middle of the recovery period.
