# Supplementary material for: The Effects of Intensive Weight Reduction on Body Composition and Serum Hormones in Female Fitness Competitors
Source: Front Physiol. 2017 Jan 10;7:689. doi: 10.3389/fphys.2016.00689 (PMC5222856; doi:10.3389/fphys.2016.00689)
Supplement: Supplementary file 2 [file Table2.PDF]

Supplementary Table 2. Average mood profile (POMS)

	Pre	Midpoint	Mid	Post
VIGOR				
Diet	3.26 ± 0.98	2.91 ± 0.85	2.93 ± 0.87	2.59 ± 0.91
Cont	2.66 ± 0.99	2.90 ± 0.89	2.74 ± 0.80	2.46 ± 0.89
CONFUSION				
Diet	1.31 ± 0.37	1.35 ± 0.48	1.56 ± 0.60	1.44 ± 0.56
Cont	1.21 ± 0.22	1.16 ± 0.15	1.44 ± 0.41	1.22 ± 0.30
DEPRESSION				
Diet	1.26 ± 0.37	1.21 ± 0.40	1.22 ± 0.37	1.35 ± 0.64
Cont	1.21 ± 0.36	1.24 ± 0.42	1.51 ± 0.73	1.26 ± 0.41
ANGER				
Diet	1.34 ± 0.42	1.51 ± 0.67	1.57 ± 0.67	1.35 ± 0.47
Cont	1.13 ± 0.24	1.18 ± 0.23	1.29 ± 0.35	1.16 ± 0.31
TENSION				
Diet	1.46 ± 0.61	1.44 ± 0.37	1.53 ± 0.56	1.46 ± 0.65
Cont	1.32 ± 0.54	1.25 ± 0.33	1.60 ± 0.63	1.34 ± 0.51
FATIGUE				
Diet	1.84 ± 0.79	2.26 ± 1.22	2.22 ± 0.95	2.15 ± 0.94
Cont	1.81 ± 0.52	1.57 ± 0.54	2.13 ± 1.05	2.01 ± 0.65

Mood was asked by the questionnaires at pre time-point, in the middle of the pre and mid timepoints (Midpoint) and the week before the mid- and post measurements. n≥17 for the diet and n=17 for the control participants from whom all the time-points were available for the analysis.
